# Müllerian Cyst in Posterior Mediastinum in a Young Woman

**DOI:** 10.4274/balkanmedj.2017.0896

**Published:** 2018-03-15

**Authors:** Jung Eun Lee, Yoon Ki Cha, Jeung Sook Kim, Jin-ho Choi, Kang Min Han

**Affiliations:** 1 Department of Radiology, Dongguk University Ilsan Hospital, Goyang, South Korea; 2 Department of Thoracic and Cardiovascular Surgery, Dongguk University Ilsan Hospital, Goyang, South Korea; 3 Department of Pathology, Dongguk University Ilsan Hospital, Goyang, South Korea

A 22-year-old female visited our hospital for a routine checkup, and a mass was incidentally found on chest radiography. She denied any medical or family history and did not complain of any symptoms. Other than obesity, physical examinations and laboratory tests were unremarkable. Contrast-enhanced chest computed tomography depicted a 2.4 cm, well-defined ovoid thin-walled cyst in the left paravertebral space adjacent to the tenth vertebra (Figure 1a, 1b). Radiologic impression indicated a benign tumour, such as a neurogenic tumour with cystic change or a neurenteric cyst. She underwent tumour resection by video-assisted thoracoscopic surgery. The mass was an ovoid unilocular cyst with a thin, translucent wall containing clear watery fluid (Figure 2a).

In the histological examination, the cyst was lined by flattened, cuboidal or ciliated columnar epithelium supported by underlying fibrous stroma (Figure 2b). The epithelial lining of the cyst consisted of tubal type epithelium, supportive of Müllerian differentiation. On immunohistochemical staining, the epithelial lining was positive for estrogen receptor, progesterone receptor, and Wilm’s Tumour 1 (Figure 2c, 2d). Finally, the cyst was diagnosed as a Müllerian cyst. She was discharged, and there was no recurrence for more than 1-year follow-up. Written informed consent was taken from the patient.

Cysts arising in the mediastinum are relatively rare, accounting for 12-30% of mediastinal masses (1). Müllerian cysts (also known as Hattori’s cysts) developing in the mediastinum are even more uncommon, with only a few cases reported since first described in 2005 (2). Most Müllerian cysts in the mediastinum have occurred in the perimenopausal period. In our case, the Müllerian cyst developed in a young woman, implying that it can be encountered at an earlier age. Obesity, gynecologic history and hormone replacement therapy have been reported to be frequent in patients with these cysts (3). The origin of mediastinal Müllerian cysts has yet to be firmly described. Several theories have been proposed to explain the histogenesis of Müllerian cysts in the mediastinum. Hattori (2) suggested that the cysts may be derived from misplaced mesothelium and mesenchyme with Müllerian characteristics similar to retroperitoneal Müllerian cysts. However, the fact that cysts in the retroperitoneum often have endocervical differentiation unlike mediastinal cysts implies that mediastinal Müllerian cysts are not simply a counterpart of retroperitoneal Müllerian cyst. Batt et al. (4) insisted that the cysts were directly originated from the primary Müllerian apparatus by adopting Ludwig’s theory of the pathogenesis of the Mayer-Rokitansky-Küster-Hauser syndrome (5). Mediastinal Müllerian cysts are seen as homogeneous, thin-walled cysts without enhancement on computed tomography. They are usually located at the posterior mediastinum, especially the paravertebral space (6). However, these findings are not specific for Müllerian cysts and are also encountered for other mediastinal cysts. Final diagnosis can be made by histological findings. Microscopically, ciliated epithelium with Müllerian differentiation confirms a diagnosis. Immunohistochemistry with positive staining for estrogen receptor, progesterone receptor, Wilm’s Tumour 1 and PAX-8 may be helpful (7).

Mediastinal Müllerian cysts follow a benign course with no reported recurrence so that surgical excision is the treatment of choice. The mediastinal Müllerian cyst is a recently established type of mediastinal cyst, which should be considered in the differential diagnosis of mediastinal cysts located in the paravertebral space of the posterior mediastinum in perimenopausal women or even in young women that are obese or have a previous gynecological history or hormonal abnormalities.

## Figures and Tables

**Figure 1:**
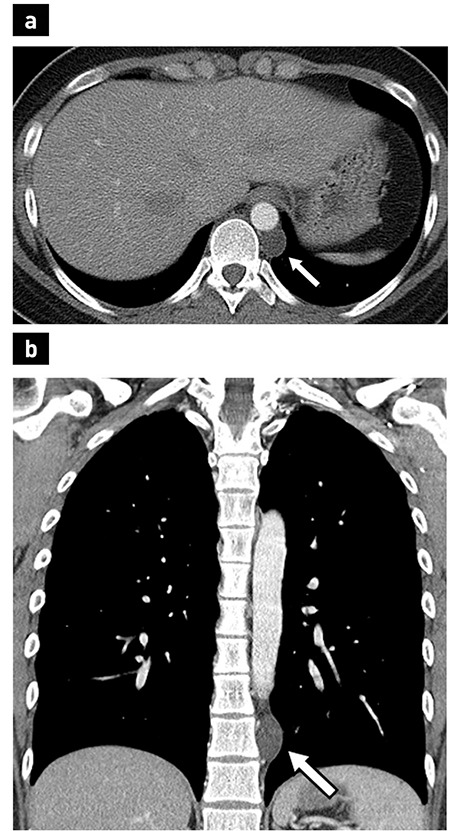
Contrast-enhanced axial (a), and coronal (b) chest computed tomography show a well-defined ovoid thin-walled cystic mass without enhancement (arrows) in the left paravertebral space adjacent to the tenth vertebra.

**Figure 2:**
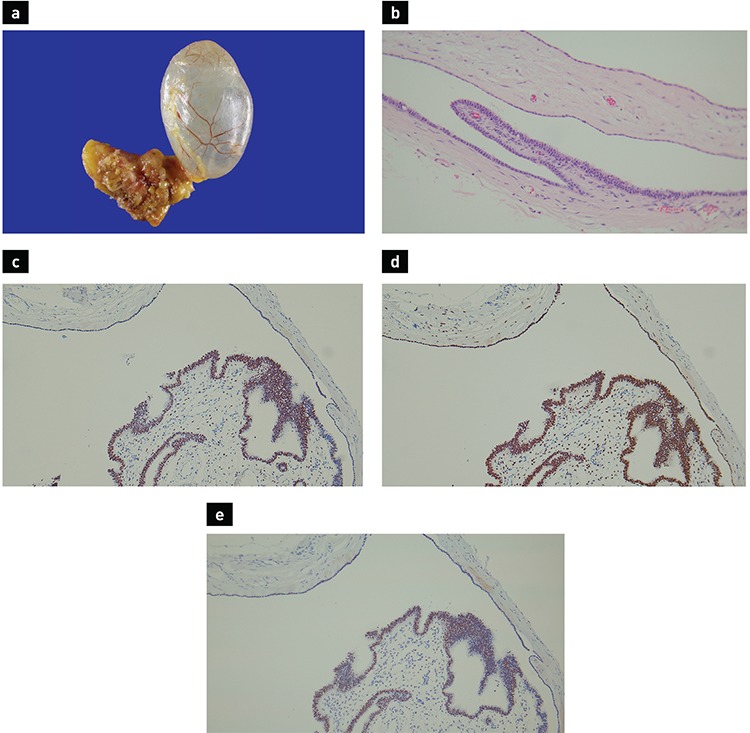
The resected specimen (a) demonstrated a cyst with a thin, translucent wall containing clear watery fluid. On histological examination, the inner surface of the cyst was lined with flattened, cuboidal or ciliated columnar epithelium, and was supported by underlying fibrous stroma (b). Immunohistochemical stainings for estrogen receptor (c), progesterone receptor (d), and Wilm’s tumour 1 (e) revealed positive nuclear staining (a: H&E original magnification x200; b: estrogen receptor x100; c: progesterone receptor x100; d: Wilm’s Tumour 1 x100).
